# Unusual Surge of Acute Hepatitis A Cases in 2016 and 2017 in Malaga, Southern Spain: Characterization and Relationship with Other Concurrent European Outbreaks

**DOI:** 10.3390/jcm12206613

**Published:** 2023-10-19

**Authors:** Paula Bardón De Tena, Silvana Teresa Tapia Paniagua, José Alberto Vico Sevilla, Encarnación Clavijo, Eduardo Martínez Manzanares, Carmen Maria Gonzalez-Domenech

**Affiliations:** 1 Microbiology Unit, Hospital de Puerto Real, 11510 Cádiz, Spain; paula.bardon@hotmail.com; 2 Department of Microbiology, Faculty of Sciences, University of Malaga, Avenue Louis Pasteur w/n, 29010 Malaga, Spain; stapia@uma.es (S.T.T.P.); eclavijo@uma.es (E.C.); emmanzanares@uma.es (E.M.M.); 3 Department of Microbiology, Faculty of Medicine, University of Granada, 18071 Granada, Spain; javs0007@correo.ugr.es; 4 Infectious Diseases and Clinical Microbiology Unit, Virgen de la Victoria Hospital, 29010 Malaga, Spain

**Keywords:** HAV, outbreak, MSM, molecular epidemiology

## Abstract

We aimed to describe the Hepatitis A virus (HAV) cases that arose in Malaga (Spain) in 2016 and 2017 when the European Centre for Disease Prevention and Control (ECDC) reported several outbreaks among men who have sex with men (MSM). Therefore, we conducted a retrospective study gathering demographic, clinical, and immunological data from the acute HAV patients attending our hospital between March 2016 and December 2017. Additionally, VP1/P2A region was amplified from serum samples, sequenced, and genotyped. We finally performed a phylogenetic analysis, including the HAV strains from the other European outbreaks. A total of 184 HAV cases were reported, with the highest number in March 2017. The cohort mostly comprised Spaniards (81.0%), males (84.8%), and MSM (72.3%), with a median age of 33.0 years (interquartile range (IQR) = 25.0–43.0). Most patients exhibited symptoms. In addition, a successful amplification and sequencing of the VP1/P2A region was performed in 25 out of 106 serum samples (23.6%). All the sequences belonged to the genotype IA, and 20 were phylogenetically related to VRD_521_2016, first described in the United Kingdom (UK). In conclusion, HAV cases emerged in Malaga in 2016 and 2017, showing an epidemic character phylogenetically related to the predominant strain first detected in the UK. Characteristics of the cohort were similar to those from the European outbreaks.

## 1. Introduction

The Hepatitis A virus (HAV) is a non-enveloped RNA virus with a positive-sense, single-stranded genome belonging to the genus *Hepatovirus* and family *Picornaviridae* [1]. HAV infection can either be asymptomatic or show a wide assortment of clinical manifestations, ranging from mild in children to acute and severe hepatitis, most common in adults. There are rarely case reports of fulminant hepatic failure and subsequent death as a complication of acute HAV infection [2]. HAV is transmitted through different routes, mainly the fecal–oral route or by consuming contaminated food and water, but also by sexual interaction [3,4,5]. 

Vaccination, sanitation, and clean drinking water are the main public health interventions underlying the decreased rate of HAV cases worldwide [6,7]. However, the drop in the HAV incidence is paradoxical: The older the infected person, the more severe the disease, increasing the morbidity [6,8]. Outbreaks occasionally appear in places of low seroprevalence but with a high proportion of susceptible individuals, mainly due to risk groups like men who have sex with men (MSM) and travelers to endemic areas [8,9,10,11]. 

Thus, the incidence of HAV in Europe remained stable one decade ago, according to figures provided by the Surveillance Atlas of Infectious Diseases https://atlas.ecdc.europa.eu/public/index.aspx (accessed 30 August 2023) from the European Centre for Disease Prevention and Control (ECDC) (i.e., 12,659 confirmed HAV cases in 2013; 14,113 cases in 2014; 12,528 in 2015). However, between 2016 and late 2017, the ECDC reported numerous HAV outbreaks, predominantly among MSM [12,13]. These outbreaks were linked to three different subgenotype IA circulating strains, VRD 521_2016, V16-25801, and RIVM-HAV16-090, first described in the United Kingdom (UK), Germany, and the Netherlands, respectively [14,15,16], but also spread to other European countries such as Spain and Italy [17,18]. Nevertheless, Spain is among the lowest incidence countries for HAV disease, with most confirmed cases related to trips and contaminated food [19].

We witnessed an increase in acute HAV cases in the middle of 2016 in Malaga, southern Spain. This rise remained during the following year [20]. We aimed to characterize this unusual increase in cases and their epidemiological link in terms of being considered one or more outbreaks. We also aimed to investigate their relationship to the other European outbreaks that emerged in the same period.

## 2. Materials and Methods

### 2.1. Study Design and Sample Collection

We collected 106 serum samples from 184 patients with acute HAV infection attended at the Virgen de la Victoria University Hospital, Málaga, southern Spain, between March 2016 and December 2017. HAV cases were defined as those with compatible symptoms (e.g., jaundice, fever, myalgias, abdominal pain, lethargy, and vomiting) and a positive confirmatory IgM antibodies HAV-specific serum test performed with VITROS™ Controls: Anti-HAV IgM (Ortho-Clinical Diagnostics™, Raritan, NJ, USA, currently partnership with Thermo Fisher Scientific™ Inc., Waltham, MA, USA). Data from patients were used under confidentiality and properly anonymized.

#### 2.1.1. Description and Statistical Analyses of the Study Population Characteristics

We conducted a retrospective, analytical, and descriptive study of the population involved, collecting demographic, clinical, and immunological information from the patients. We performed a statistical analysis of the variables with the software SPSS v16.1. Before the descriptive analysis, we studied whether the distribution of the corresponding variable in the cohort was adjusted to normality. The Wilcoxon non-parametric test was used for quantitative variables following a non-normal distribution. The comparison of proportions was conducted by using the bilateral Fisher test. The association or independence of quantitative variables (normally distributed) with a dichotomous category was assessed in the two categories with the Student’s t-test. In all cases, statistical significance was set at *p* < 0.05. The MSM status was defined as self-identifying as MSM or reporting sexual contact with another man.

#### 2.1.2. Ethical Concerns

Because of the emergency healthcare, participants were not asked to provide informed consent. However, data were anonymously analyzed, and no personal information has been depicted in this manuscript. Moreover, ethical approval was obtained as described in the Ethical Approval statement section.

#### 2.1.3. HAV Amplification and Sequencing

RNA was extracted from 300 µL of serum samples using the Speedtools RNA virus extraction kit (Biotools B&M Labs S.A., Madrid, Spain). Then, HAV-RNA was reverse transcribed to cDNA using the Maxima First Strand cDNA Synthesis kit (Thermo Fisher Scientific™ Inc., USA), as specified by the manufacturers. Later, we amplified the VP1/P2A region by using the nested polymerase chain reaction (PCR). The first PCR amplification was performed following Michaelis’s protocol for the first-round conditions [21], with the same specific primers used (HAV 6.1, 5′-TAT GCY ITI TCW GGI GCI YTR GAY GG-3′; HAV 10, 5′-TCY TTC ATY TCW GTC CAY TTY TCA TCA TT-3′, 614 nucleotides (nt)). Briefly, we performed a 10-min initial enzyme activation step at 95 °C, and 45 cycles of 95 °C for 15 s and 60 °C for 1 min. In both nested PCR reactions, we used AccuStart II PCR SuperMix (VWR International, LLC., Radnor, PA, USA), a ready-to-use reaction cocktail for routine PCR amplification. The second PCR was performed using 10 µL from the first round of PCR as a template, following the protocol from HAV Net, the National Institute for Public Health and the Environment (RIVM) [22]. The pair of specific primers for the internal VP1/2A region were HAV8.2-F (5′-GGATTGGTTTCCATTCARATTGCNAAYTA-3′) and HAV11-R (5′-CTGCCAGTCAGAACTCCRGCWTCCATYTC-3′). This second amplification also comprised an initial step of 6 min at 95 °C and then 40 cycles of 95 °C for 30 s, 60 °C for 20 s, and 72 °C for 15 s. The resulting sequence was 507 nt, with a size confirmation for all the PCR products by agarose gel electrophoresis. 

Sequencing was outsourced and performed by Macrogen Laboratory Spain (Madrid, Spain), using as the initiation primer the previously mentioned HAV8.2-F. We amplified and sequenced the VP1/P2A junction region (over 500 nt, positions 2923 to 3440 in the reference HAV strain, Genbank accession number NC_001489). 

#### 2.1.4. Molecular Genotyping of HAV Samples

Using the VP1/P2A region sequence, we identified the Hepatitis A virus genotype by means of Hepatitis A Virus Genotyping Tool Version 1.0 “https://www.rivm.nl/mpf/typingtool/hav/ (accessed 5 August 2021), created by HAVnet (RIVM). 

#### 2.1.5. Phylogenetic Analysis of HAV Sequence Data

The relationship among our HAV sequences—and compared to the epidemic outbreaks in other European countries between 2016 and 2018—was first characterized by a preliminary phylogenetic analysis. For such a purpose, we included the sequence V16-25801 (GenBank acc. Numb. LT796556.1) first reported in Germany [16], and the sequences BCN17_HAV_01 (GenBank acc. Numb. MF805869.1) and BCN17/HAV/06 (GenBank acc. Numb. MF805872.1), identical to the sequences VRD_521_2016 and RIVM-HAV16-090, originally linked to the epidemic clusters in the UK and the Netherlands, respectively [17]. Reference sequences were retrieved from GenBank and included as well Genotype IA: EU131373; AB020565.1; Genotype IB: M14707; DQ646426; NC001489; AF314208; Genotype IIA: AY644676; Genotype IIB: AY644670; Genotype IIIA: AJ299464; DQ991030; AB279733; and Genotype IIIB: AB279735; AB425339; AB258387. 

All the sequences were aligned by using ClustalX [23] and manually edited by Jalview [24]. All the HAV sequences collected from the literature that did not span the VP1X2A junction region were removed from the final alignment, with an average overall length of 500 nt. Then, we reconstructed a phylogeny with the maximum-likelihood (ML) method using MEGA software v11 [25]. We applied the TN93 substitution model as the best one according to the Akaike Information Criterion (AIC) determined by FindModel, also included in MEGA [25]. The reliability of each grouping on the resulting tree was assessed from its bootstrap resampling value based on 1000 iterations (>70% considered significant). In addition, the phylogeny obtained was confirmed by Bayesian analysis if the associated posterior probability (pp) was ≥0.9. The Bayesian approach was performed using MrBayes v3.2 program [26] and then visualized with the graphical viewer FigTree v1.4.3. MrBayes analysis was also performed with the evolutionary model GTR + I + G for 20 million generations, sampled every 100 generations and with a 25% default burn-in value for diagnostics.

## 3. Results

Characterization of HAV Cases in Malaga and Phylogenetic Relationship with European Outbreaks

**Figure 1:**
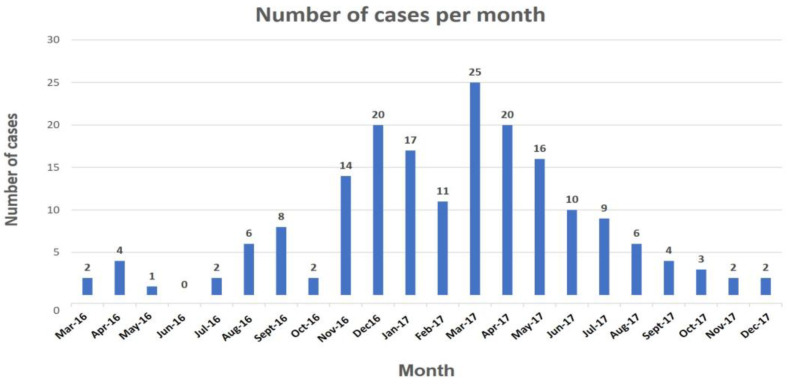
Cases of HAV over time detected in the Virgen de la Victoria Hospital (Malaga), from the first cases reported in March 2016 until the end of the study period (2017).

From March 2016 until December 2017, 184 cases of Hepatitis A were reported in the Virgen de la Victoria University Hospital. Serum samples were available for 106 of them (57.6%). The highest prevalence of cases was found in March 2017, with 25 patients (13.6%) (Figure 1). 

A detailed description of the main demographic, biochemical, and clinical data from the entire cohort can be found in Supplementary Tables S1 and S2. As observed, the median age of the cohort was 33.0 years (interquartile range (IQR) = 25.0–43.0), most of the patients are from Spain (81. 0%, *p* < 0.001), males (84.8%, *p* < 0.001) and MSM (72.3%, *p* < 0.001). Almost half of them were attended in the Emergency Unit of the hospital, and they mostly showed symptomatology. In that sense, patients exhibited variable symptoms: Choluria (64.7%), jaundice (57.6%), fever (53.8%), and sickness (53.3%) were the most frequent clinical manifestations, whereas acholia (27.7%), diarrhea (18.5%), and free intraperitoneal fluid (9.8%) were the less common ones (Supplementary Table S1). Thirty out of 184 (17.4%) showed a coinfection, with the highest rate for HIV (8.7%), syphilis (3.8%), and both infections together (2.7%). Biochemical parameters of liver function (alkaline phosphatase, C-reactive protein, bilirubin, lactate dehydrogenase, and the three transaminases) were extremely impaired (Supplementary Table S2). Finally, the hemogram test was normal, except for prothrombin time, slightly lower than reference values. 

**Figure 2:**
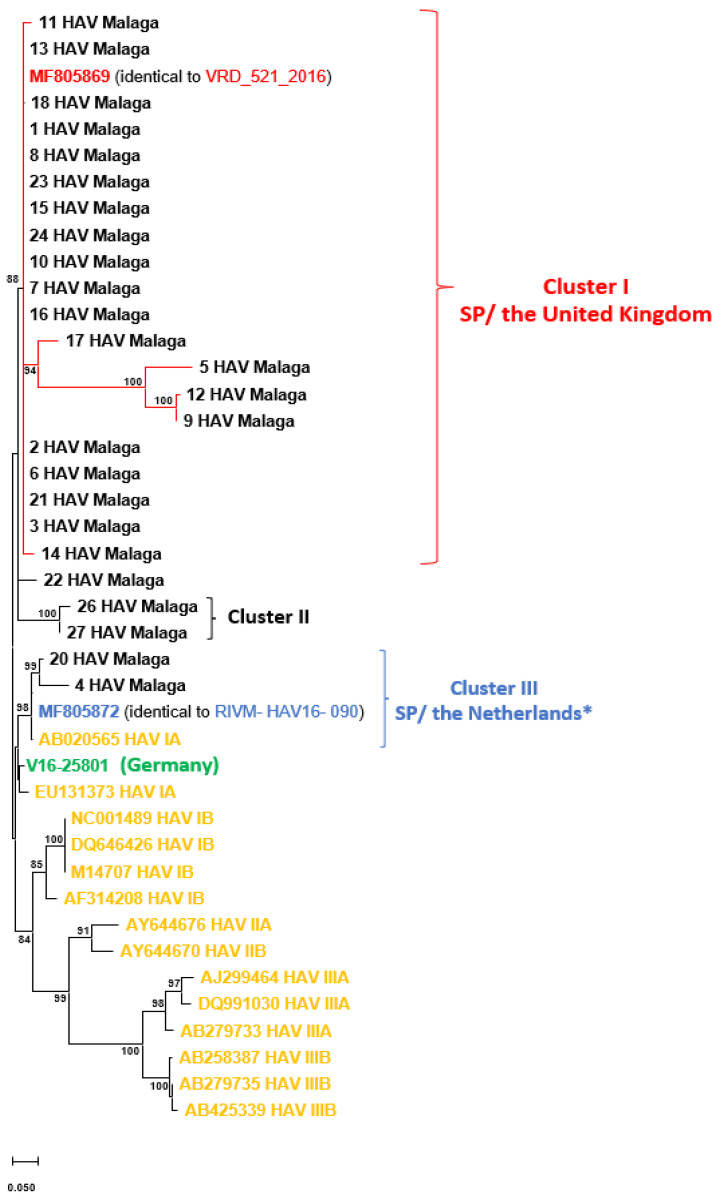
Phylogenetic inference based on VP1/P2A region (approx. 510 nt) from sequences obtained in HAV patients from our area between 2016 and 2017. The phylogeny was derived from the Maximum Likelihood method with bootstrap values as a measure of branch support. The analysis was conducted in MEGA v11. Each sample ID denoted an HAV strain from the Malaga area. Genotypes other than IA were used as out-groups and are colored in light orange (Roman numerals designate each genotype). Only bootstrap proportions >70% are shown. Prototype strains from European outbreaks (VRD_521_2016, V16-25801, and RIVM-HAV16-090) (or identical ones) and country of origin are indicated in different colors. * This cluster was not confirmed by Bayesian Analysis.

In addition, we amplified the VP1/P2A region sequence in 27 out of 106 serum samples (25.5%), but two sequences were discarded as suggestive of contamination. Thus, 25 sequences (13.6% of the total cases reported) were finally available for further phylogenetic analysis to confirm the existence of an outbreak. Among them, 6 out of 25 (24%) had also been obtained in March 2017, and another 4 (16%) in June 2017, whereas the rest of the collection dates were spread throughout the study period. The sequences analyzed (n = 20) belonged to the genotype IA, according to the HAV Genotyping Tool Version 1.0 by HAVnet (RIVM); the remaining five sequences could not be genotyped with this tool despite spanning the correct region (Supplementary material S1). However, the genotype assignment might also be genotype IA, as supported by our phylogenetic relationship with all the reference strains for each genotype (Figure 2 and Figure S1).

A phylogenetic analysis confirmed the relationship between the numerous HAV cases that suddenly arose in our area and the European outbreaks in the same period (Figure 2). Thus, 20 out of 25 study sequences, range sampled from April 2016 to October 2017 (Supplementary Table S3), conformed a well-defined monophyletic cluster (Cluster I) in the ML tree (bootstrap = 88%), which was related to BCN17/HAV/01 (GenBank accession number MF805869), a HAV strain identical to VRD_521_2016, first isolated from a patient in the UK [14,17]. The presence of this local transmission cluster related to one of the European strains was also confirmed by a Bayesian approach with an associated posterior probability (pp) of 0.99 (Figure S1). We inferred the phylogeny with the TN93 model using a Gamma distribution (+G) with five categories, according to results from FindModel (shown in Figure S2). In addition, another transmission cluster, Cluster III, comprising two local strains (20 and 4 HAV Malaga) linked to the original Dutch HAV strain, could not be confirmed by Bayesian analysis (Figure S1). Nevertheless, if we forced the topology with constraint command, the grouping of these three strains would be supported statistically (Supplementary material S2). Moreover, another two sequences, 26 and 27 HAV Malaga strains, were clustered together (Cluster II, bootstrap = 100%) but without any phylogenetic relationship to the European strains. In addition, one sequence was more phylogenetically separated from the rest (22 HAV Malaga strain). 

Furthermore, as shown in Table 1, Cluster I SP/UK was mostly composed of Spanish males (80%), with a median age of 32.5 years (27.8–49.8). Elucidating the risk behavior was impossible for a high percentage of Cluster I members (60%), but a quarter of the total self-reported as MSM. In addition, 4 out of 20 (15%) showed a coinfection (HIV n = 3; HIV + syphilis n = 1). All the members except two showed hepatitis typical symptoms, such as choluria (80%), fever (65%), sickness (60%), and jaundice (55%) as the predominant ones. Unlike the general cohort, a higher percentage of members belonging to Cluster I had to be hospitalized (35%).

The biochemical profile of the HAV patients from Cluster I (Table 2) is similar to that of the entire cohort (Supplementary Table S2), highlighting raised alkaline phosphatase, total serum bilirubin, and especially the extremely elevated serum glutamic oxaloacetic transaminase (SGOT), serum glutamic pyruvic transaminase (SGPT), and serum gamma-glutamyl transferase (SGGT) levels in all cases.

## 4. Discussion

Between 2016 and 2017, we observed an unusual increase in acute HAV cases in the Malaga area, southern Spain; almost half were MSM. During the same period, the ECDC reported several outbreaks, also predominantly among MSM. They were linked to three different subgenotype IA circulating strains: VRD 521_2016, V16-25801, and RIVM-HAV16-090, first described in the United Kingdom, Germany, and the Netherlands, respectively [14,15,16]. Hence, we present here a detailed characterization of the striking increase in cases that arose in our area during these two years and an assessment of their potential link to the European outbreaks.

Sexual but not foodborne transmission was the most likely cause underlying the spread of the European outbreaks [12,13]. Our cohort was half composed of MSM (45.1%) too, suggesting a similar risk behavior as the origin of the observed unusual figure of HAV patients in our area during the study period. Nevertheless, the magnitude and fast dissemination of cases were such that they finally affected the general population, as found in other European places [27,28,29]. Thus, France reported a fivefold increase in 2017 than observed in previous years regarding HAV-RNA-positive cases among blood donors, and only one was self-reported as MSM [27]. All those cases, except one, were also genotype IA, the same to which the European outbreak-associated strains belonged and that we found in our genotyping. Moreover, the extent of the outbreaks occurring in Europe in 2016 and 2017 posed the final implication of other transmission mechanisms besides the sexual one, mainly involving infected food handlers [28,29]. 

Regarding the timeframe, the rise of Hepatitis A cases occurring in Malaga hesitantly started at the beginning of 2016, with two peaks (December 2016 and March of 2017). The zenith of the other European HAV outbreaks also overlapped in time with the larger emergence of cases in Malaga [12,13,14,15,16]. Among the concurred epidemics, we also must highlight another outbreak that emerged in Spain and is epidemiologically related to the strain VRD_521_2016, the predominant in the UK outbreak [12,13,14,17]. As observed in Catalonia, most HAV cases in Malaga were also linked to the most common circulating strain in the UK. The previous epidemiological studies pointed to exportation to the UK from multiple Spanish regions and further dissemination within the MSM population in the UK [12,13,14]. Our study infers the phylogenetic relationship between cases from another additional area in Spain, Malaga, and the HAV epidemic spread in the UK in 2016 and 2017 without deepening in other phylodynamic aspects. Finally, the phylogenetic relationship of other local cases with the two remaining strains circulating in the European outbreaks, V16-25801 and RIVM-HAV16-090, was absent for the former and scarce for the latter, with only two sequences grouped with the Dutch strain. Hence, the presence of different clusters, besides unlinked sequences, may represent separate routes of introduction of HAV in our area for the study period. 

The demographic, clinical, and biochemical characteristics of HAV cases from our cohort were similar to those observed in the aforementioned outbreaks. However, we might emphasize a less severe manifestation of the disease, with a lower percentage of hospitalizations and a lower increase in serum transaminase levels (although still very high compared to the normal values) than that found in the outbreak of Catalonia, for instance [17]. 

The main limitation of our study concerns the inability to gather demographic and clinical information from our cohort and obtain serum samples from most of the patients because, due to the nature of the outbreak, most cases were attended at the emergency service. This condition sometimes led to occasional support from different hospital units without further follow-up.

## 5. Conclusions

In conclusion, the increase in HAV cases that emerged in Malaga in 2016 and 2017 possessed a phylogenetic relationship, mainly with the predominant strain detected in the UK in that period, VRD 521_2016. The characteristics of the cohort were similar to those from the European outbreaks, with a predominance of young MSM adults. 

## Figures and Tables

**Table 1:** Summary of the main demographic and clinical characteristics of the study population.

Variables		SP/UK Cluster	*p-*Value
**Number of patients**		20	
**Age (years)**		32.5 (27.8–49.8)	
**Sex**	**Male**	16 (80)	<0.001
	**Female**	4 (20)	
**Origin**	**Spanish**	15 (75)	<0.001
	**Immigrants**		
	South American	1 (5)	
	European countries *	3 (15)	
	**Unknown**	1 (5)	
**Risk behaviour**	**MSM**	5 (25)	<0.001
	**HTX**	1 (5)	
	**Piercing, tattoos**	0 (0)	
	**Trip to endemic country**	0 (0)	
	**Family contact with HAV+**	1 (5)	
**Coinfection**	**No**	16 (80)	<0.001
	**Yes**	4 (20)	
	HIV	3 (15)	
	HIV + Syphilis	1 (5)	
**Assistance department**	**Primary care**	4 (20)	
	**Emergency department**	8 (40)	
	**Out-Patient Consultation**	1 (5)	
	**In-patient stay**	5 (25)	
	**Other hospital department**	2 (10)	
**Clinical symptoms**	**No**	2 (10)	<0.001
	**Yes**	18 (90)	
	Diarrhea	3 (15)	
	Free intraperitoneal fluid	1 (5)	
	Choluria (bile in urine)	16 (80)	
	Acholia (white stools)	4 (20)	
	Jaundice	11 (55)	
	Fever	13 (65)	
	Sickness	12 (60)	
	Vomiting	9 (45)	
**Outcome**	**Hospitalization**	7 (35)	
	**Hospitalization in ICU**	0 (0)	

The quantitative age variable is expressed as median with the interquartile range (IQR), whereas the qualitative variables are expressed as n (%). * Other than Spain. HIV: Human Immunodeficiency Virus; HTX: heterosexual transmission; ICU: intensive care unit; MSM: Men who have sex with men.

**Table 2:** Baseline biochemical and hematological characteristics of the clustered population.

Variables	SP/UK Cluster	Reference Ranges
	Mean ± SD	
**Total of patients**	20	
***Biochemical parameters***		
**Albumin (g/dL)**	3.4 ± 0.6	3.5–5.5
**Alkaline phosphatase (IU/L)**	214.8 ± 106.9	20–125
**C-reactive protein (mg/dL)**	13.1 ± 9.0	<0.5
**Direct bilirubin (** **mg/dL)**	5.3 ± 2.7	0–0.3
**Direct bilirubin zenith (mg/dL)**	6.1 ± 2.1	
**Total bilirubin (** **mg/dL)**	5.9 ± 3.8	0.3–1.2
**Total bilirubin zenith (mg/dL)**	6.6 ± 3.3	
**SGGT (IU/L)**	347.1 ± 186.9	8–78
**SGOT (IU/L)**	1382.4 ± 1000.0	0–35
**SGOT zenith (IU/L)**	1732.4 ± 1178.2	
**SGPT (IU/L)**	1812.3 ± 1236.8	0–35
**SGPT zenith (IU/L)**	2068.3 ± 1710.0	
**Lactate dehydrogenase (IU/L)**	495.5 ± 246.8	60–160
**Total cholesterol (mg/dL)**	135.5 ± 65.9	140–200
**Serum creatinine (mg/dL)**	0.9 ± 0.2	0.7–1.3
**Urea (mg/dL)**	25.2 ± 12.5	20–50
***Hemogram***		
**Prothrombin time (%)**	74.0 ± 18.2	80–120
**International Normalized Ratio (INR)**	1.2 ± 0.2	0.8–1.2
**Red blood cell count (×10^6^/mL)**	5.0 ± 0.5	3.6–6.0
**Hematocrit (%)**	42.5 ± 3.8	35–46
**Leukocyte count (** **×10^3^/mL)**	5.5 ± 1.6	4–11.5
**Platelet count (** **×10^3^/mL)**	205.9 ± 42.0	140–450

SGGT, serum gamma-glutamyl transferase; SGOT, serum glutamic oxaloacetic transaminase; SGPT, serum glutamic pyruvic transaminase.

## Data Availability

Newly derived sequences are deposited and publicly available in GenBank (http://www.ncbi.nlm.nih.gov/genbank) under accessions numbers OQ722120-OQ722144.

## Supplementary Materials

The following supporting information can be downloaded at https://www.mdpi.com/article/10.3390/jcm12206613/s1: Figure S1. Bayesian phylogenetic inference obtained by MrBayes v3.2 program. Reference sequences appear with their corresponding accession numbers, while study patients clustered with the English variant are shaded in red. Numbers near each node show posterior probabilities. Genotypes other than IA were colored in light orange (Roman numerals designate each genotype); Figure S2. Maximum likelihood fits of 24 different nucleotide substitution models. The best substitution model was chosen according to the lowest Akaike Information Criterion (AIC) score as the selection criterion; Supplementary Table S1. Summary of the main demographic and clinical characteristics of the study population; Supplementary Table S2. Baseline biochemical and hematological characteristics of the study population; Supplementary Table S3. Sampling date of the strains belonging to the Cluster I, linked to VRD_521_2016 strain; Supplementary material S1. Molecular Genotyping of HAV Samples; and Supplementary material S2. Different topologies clarified by further Bayesian analysis.
