# Supplementary material for: Unusual Surge of Acute Hepatitis A Cases in 2016 and 2017 in Malaga, Southern Spain: Characterization and Relationship with Other Concurrent European Outbreaks
Source: J Clin Med. 2023 Oct 19;12(20):6613. doi: 10.3390/jcm12206613 (PMC10607832; doi:10.3390/jcm12206613)
Supplement: Supplementary file 1 [file jcm-12-06613-s001.zip › Supplementary material S2.pdf]

DEALING WITH DIFFERENT TOPOLOGIES (ML tree -Fig. 2- and Bayesian Inference -)

We employed the following commands to test the two topologies arisen by the two phylogenetic approaches and comprising the three sequences:

MrBayes > execute archivo.nex
MrBayes > constraint clustering = 20_HAV_Malaga 4_HAV_Malaga MF805872 (identical to RIVM- HAV16- 090)
MrBayes> constraint noclustering negative = 20_HAV_Malaga 4_HAV_Malaga MF805872 (identical to RIVM- HAV16- 090)

Hypothesis 1: topology considering the clustering among 20 and 4 HAV Malaga strains with the original Dutch HAV strain RIVM-HAV16- 090

Run	Arithmetic mean	Harmonic mean
1	-3441.03	-3467.47
2	-3437.91	-3470.62
TOTAL	-3438.56	-3469.97

Hypothesis 2: topology considering an independent clustering among 20 and 4 HAV Malaga strains, and the original Dutch HAV strain RIVM-HAV16- 090

Run	Arithmetic mean	Harmonic mean
1	-3435.67	-3475.03
2	-3439.09	-3467.36
TOTAL	-3436.33	-3474.34

$2log(B_{12}) = 2 * (-3469.97 - (-3474.34) = 8.74$, where B_{12} is the Bayes factor. According to the following table:

$2\log_e(B_{12})$	B_{12}	Evidence against H_2
0 to 2	1 to 3	Not worth more than a bare mention
2 to 6	3 to 20	Positive
6 to 10	20 to 150	Strong
>10	>150	Very strong

Interpretation of the Bayes factor (*B*12). Based on (Kass and Raftery, 1995).

Therefore, there is strong evidence against the second hypothesis (compared to H_1), that is, against a separate branch among local strains (20 HAV Malaga and 4 HAV Malaga) and the Dutch strain.
