# Supplementary material for: Unusual Surge of Acute Hepatitis A Cases in 2016 and 2017 in Malaga, Southern Spain: Characterization and Relationship with Other Concurrent European Outbreaks
Source: J Clin Med. 2023 Oct 19;12(20):6613. doi: 10.3390/jcm12206613 (PMC10607832; doi:10.3390/jcm12206613)
Supplement: Supplementary file 1 [file jcm-12-06613-s001.zip › Supplementary Table S1.pdf]

Supplementary Table S1. Summary of the main demographic and clinical characteristics of the study population.

Variables		*Total*	*p-value*
**Number of patients**		184	
**Average age in year (SD)**		33(25.0-43.0)	
**Sex**	**Male**	156(84.8)	<0.001
	**Female**	28(15.2)	
**Origin**	**Spanish**	149(81.0)	<0.001
	**Inmigrants**		
	South American	8(4.3)	
	European countries*	12(6.5)	
	African	2(1.1)	
	Asian	1(0.5)	
	**Unknown**	12(6.5)	
**Risk behaviour**	**MSM**	83(45.1)	<0.001
	**HTX**	2(1.1)	
	**Piercing, tatoos**	1(0.5)	
	**Trip to endemic country**	2(1.1)	
	**Familiar contact with HAV+**	10(5.4)	
	**Food/water intake**	1(0.5)	
	**Others**	56(30.4)	
	**Unknown**	29(15.8)	
**Unique HAV infection (without co-infection)**	**Yes**	152(82.6)	<0.001
	**No**	32(17.4)	
	HIV	16(8.7)	
	HVB	1(0.5)	
	HIV+HBV	1(0.5)	
	HVC	1(0.5)	
	Syphilis	7(3.8)	
	Syphilis+HIV	5(2.7)	
	Syphilis+HIV+HBV	1(0.5)	
**Assistance department**	**Primary care**	41(22.3)	<0.001
	**Emergency department**	86 (46.7)	
	**Out-Patient Consultation**	18(9.8)	
	**In-patient stay**	32(17.4)	
	**Other hospital department**	7(3.8)	
**Clinical symptoms**	**Yes**	155(84.2)	<0.001
	**No**	4(2.2)	
	**Unknown**	25(13.6)	
**Outcome**	**Hospitalization**	46(13.6)	0.540
	**Hospitalization in ICU**	1(0.5)	<0.001

The quantitative variable of age is expressed as median with IQR, whereas the qualitative variables as n (%).

*Other than Spain

HIV: Human Immunodeficiency Virus; HVB: Hepatitis virus B; HTX: heterosexual transmission; HVC: Hepatitis virus C; ICU: intensive care unit; MSM: Men who have sex with men
