# Supplementary material for: Unusual Surge of Acute Hepatitis A Cases in 2016 and 2017 in Malaga, Southern Spain: Characterization and Relationship with Other Concurrent European Outbreaks
Source: J Clin Med. 2023 Oct 19;12(20):6613. doi: 10.3390/jcm12206613 (PMC10607832; doi:10.3390/jcm12206613)
Supplement: Supplementary file 1 [file jcm-12-06613-s001.zip › Supplementary Table S2.pdf]

Supplementary Table S2. Baseline biochemical and haematological characteristics of the study population.

Variables	Mean ± SD	Reference ranges
**Total of patients**	184	
Biochemical parameters		
**Albumin (g/dL)**	3.4 ± 0.5	3.5 - 5.5
**Alkaline phosphatase (UI/L)**	198.7 ± 143.7	20 - 125
**C-reactive protein (mg/dL)**	12.5 ± 12.6	< 0.5
**Direct bilirubin (mg/dL)**	5.4 ± 2.5	0 - 0.3
**Direct bilirubin zenith (mg/dL)**	5.7 ± 2.5	
**Total bilirubin (mg/dL)**	5.7 ± 3.8	0.3–1.2
**Total bilirubin zenith (mg/dL)**	6.7 ± 5.7	
**SGGT (UI)**	275.5 ± 246.3	8-78
**SGOT (UI)**	1305.8 ± 1331.1	0 - 35
***S*GOT zenith (UI)**	1616.9 ± 1693.5	
**SGPT (UI)**	1630.4 ± 1658.4	0 - 35
**SGPT zenith (UI)**	1716.3 ± 1774.4	
**Lactate dehydrogenase (U/L)**	530.1 ± 460.3	60-160
**Total cholesterol (mg/dl)**	142.8 ± 62.3	140 - 200
**Serum creatinin (mg/L)**	0.9 ± 0.2	0.7-1.3
**Urea (U/L)**	25.3 ± 9.8	20 - 50
Haemogram		
**Prothrombin time (%)**	73.5 ± 18.8	80 - 120
**International Normalized Ratio (INR)**	1.2 ± 0.2	0.8 - 1.2
**Red blood cells count (cells/L)**	5.0 ± 0.7	3.6 - 6.0
**Hematocrit (%)**	43.5 ± 4.6	35 - 46
**Leukocytes count (cells/L)**	6.3 ± 2.5	4 - 11.5
**Platelets count (cells/L)**	230.6 ± 83.6	140 - 450

SGGT, serum gamma-glutamyl transferase; SGOT, serum glutamic oxaloacetic transaminase; SGPT, serum glutamic pyruvic transaminase;
