# Supplementary material for: Unusual Surge of Acute Hepatitis A Cases in 2016 and 2017 in Malaga, Southern Spain: Characterization and Relationship with Other Concurrent European Outbreaks
Source: J Clin Med. 2023 Oct 19;12(20):6613. doi: 10.3390/jcm12206613 (PMC10607832; doi:10.3390/jcm12206613)
Supplement: Supplementary file 1 [file jcm-12-06613-s001.zip › Supplementary Table S3.pdf]

Supplementary Table S3. Sampling date of the strains belonging to the Cluster I, linked to VRD_521_2016 strain.

Strain	Sampling date (DD/MM/YYYY)
**1_HAV_Malaga**	12/12/2016
**2_HAV_Malaga**	11/04/2017
**3_HAV_Malaga**	22/05/2017
**5_HAV_Malaga**	02/06/2017
**6_HAV_Malaga**	24/03/2017
**7_HAV_Malaga**	05/04/2017
**8_HAV_Malaga**	05/10/2017
**9_HAV_Malaga**	04/04/2017
**10_HAV_Malaga**	22/11/2016
**11_HAV_Malaga**	05/12/2016
**12_HAV_Malaga**	01/06/2017
**13_HAV_Malaga**	01/03/2017
**14_HAV_Malaga**	09/03/2017
**15_HAV_Malaga**	03/03/2017
**16_HAV_Malaga**	02/05/2017
**17_HAV_Malaga**	20/01/2017
**18_HAV_Malaga**	11/04/2016
**21_HAV_Malaga**	28/06/2017
**23_HAV_Malaga**	31/07/2017
**24_HAV_Malaga**	30/03/2017
