# Supplementary figures and images for: Unusual Surge of Acute Hepatitis A Cases in 2016 and 2017 in Malaga, Southern Spain: Characterization and Relationship with Other Concurrent European Outbreaks
Source: J Clin Med. 2023 Oct 19;12(20):6613. doi: 10.3390/jcm12206613 (PMC10607832; doi:10.3390/jcm12206613)

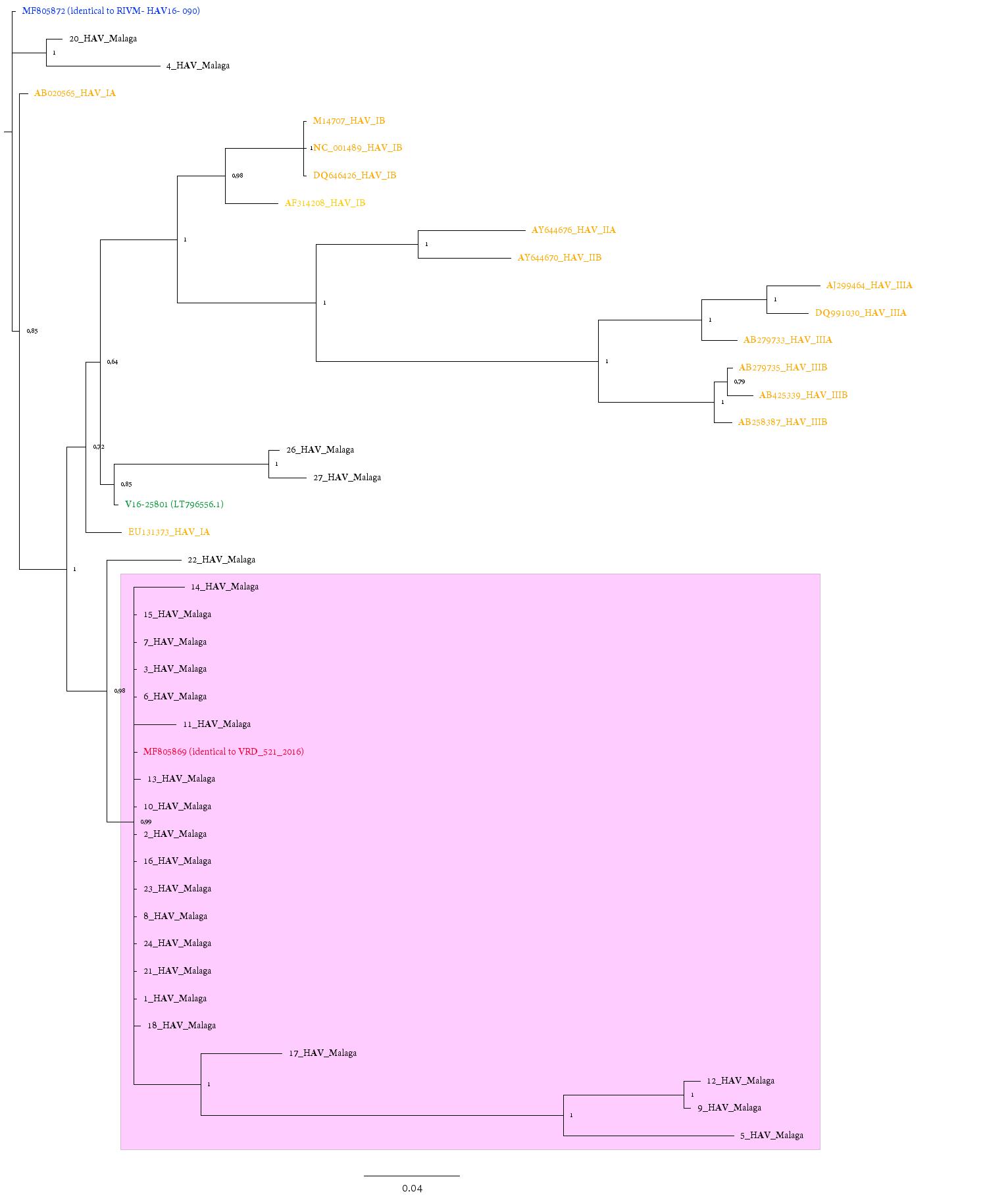

Supplement: Supplementary file 1 [file jcm-12-06613-s001.zip › Figure S1.jpg]

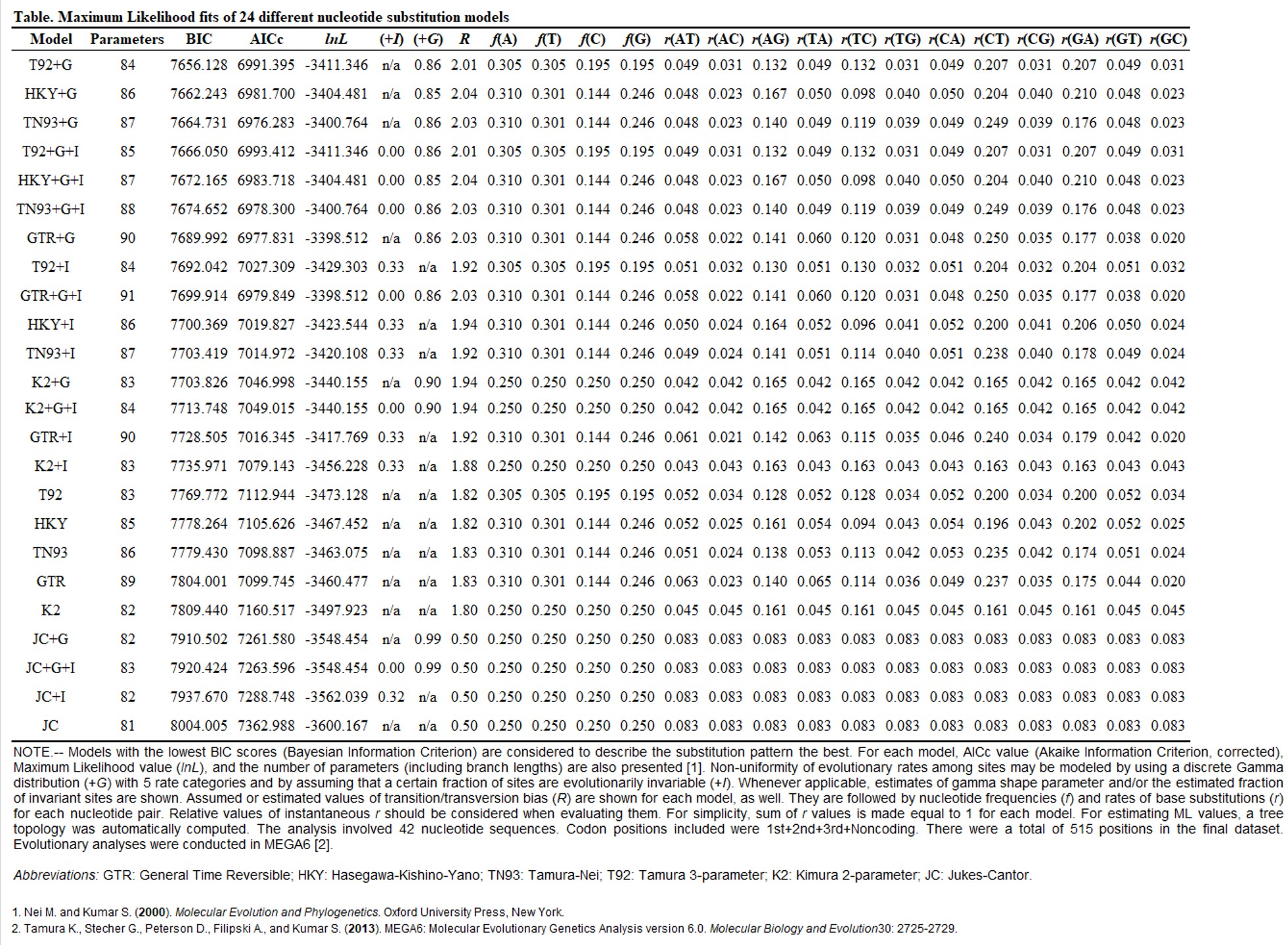

Supplement: Supplementary file 1 [file jcm-12-06613-s001.zip › Figure S2.jpg]
